# Supplementary material for: Comparative Analysis of Human and Animal E. coli: Serotyping, Antimicrobial Resistance, and Virulence Gene Profiling
Source: Antibiotics (Basel). 2022 Apr 21;11(5):552. doi: 10.3390/antibiotics11050552 (PMC9137549; doi:10.3390/antibiotics11050552)
Supplement: Supplementary file 1 [file antibiotics-11-00552-s001.zip › antibiotics-1678782-supplementary.pdf]

antibiotics

MDPI

Supplementary data

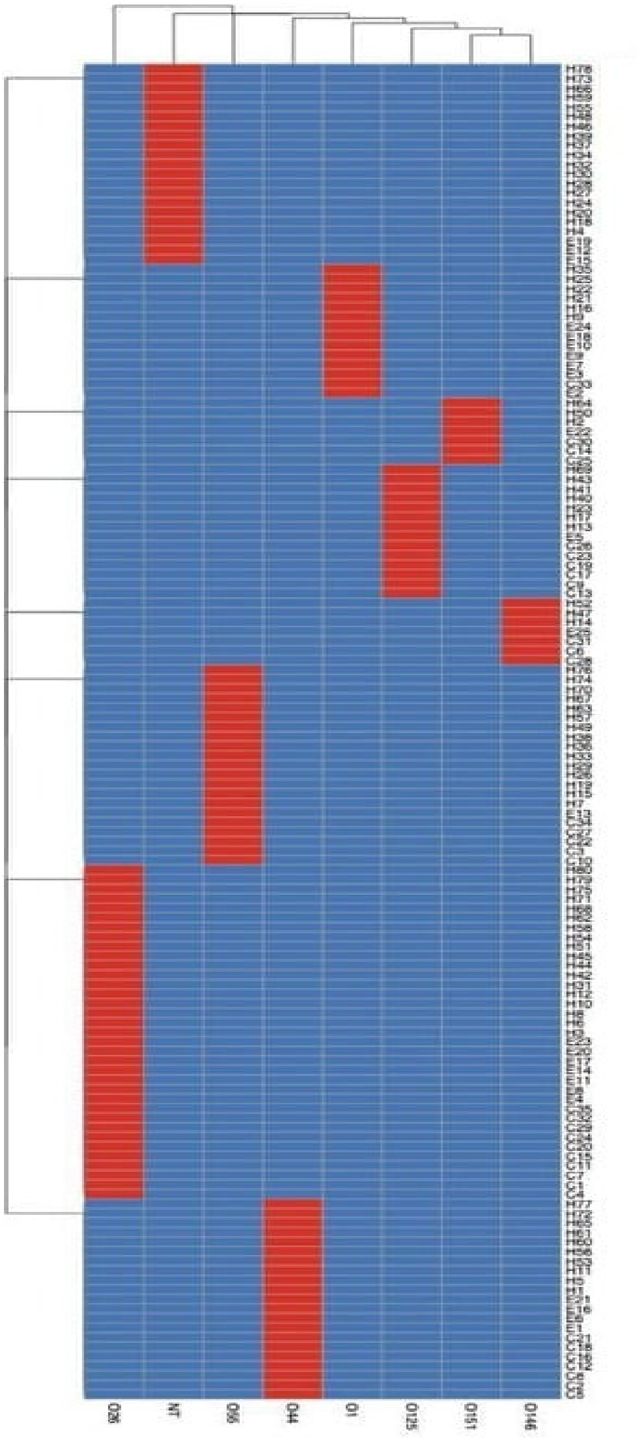

Figure S1: Heat map showing the occurrence of serotypes in the studied diarrheagenic *E. coli* isolates. In the heat map, red and blue colors refer to the presence and absence of a particular serotype, respectively. The dendogram represents the hierarchical clustering of the isolates and the serotypes. The code numbers on the right of the heat map refer to the isolate numbers from human (H), cow (C) and equine (E) sources.

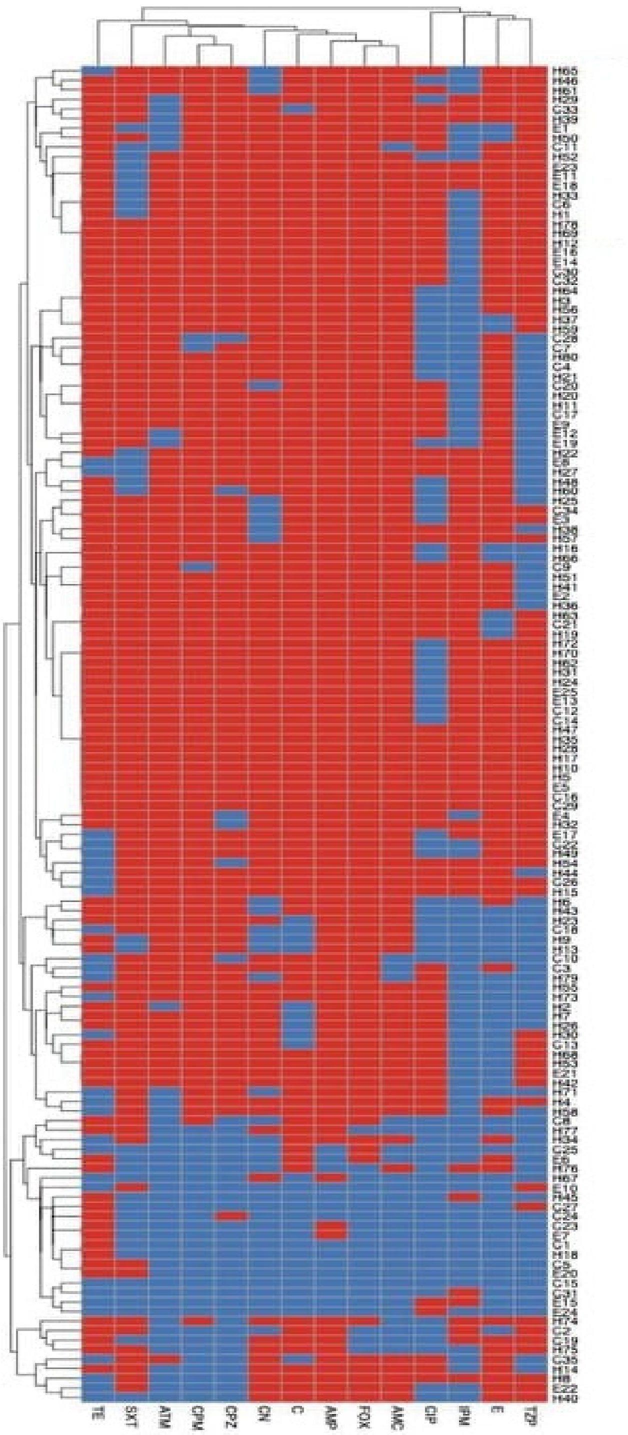

Figure S2: Heat map showing the occurrence of antimicrobial resistances in the studied diarrheagenic *E. coli* isolates. In the heat map, red and blue colors refer to the resistance and sensitivity to a particular antimicrobial agent, respectively. The dendogram represents the hierarchical clustering of the isolates and antimicrobial resistances. The code numbers on the right of the heat map refer to the isolate numbers from human (H), cow (C) and equine (E) sources.

AMP: ampicillin, AMC: amoxicillin/clavulanic acid, ATM: aztreonam, FOX: cefoxitin, CPZ: cefoperazone, CPM: cefepime, TZP: piperacillin/tazobactam, IPM: imipenem, C: chloramphenicol, E: erythromycin, CN: gentamycin, CIP: ciprofloxacin, SXT: sulfamethoxazole/trimethoprim, TE: tetracycline.

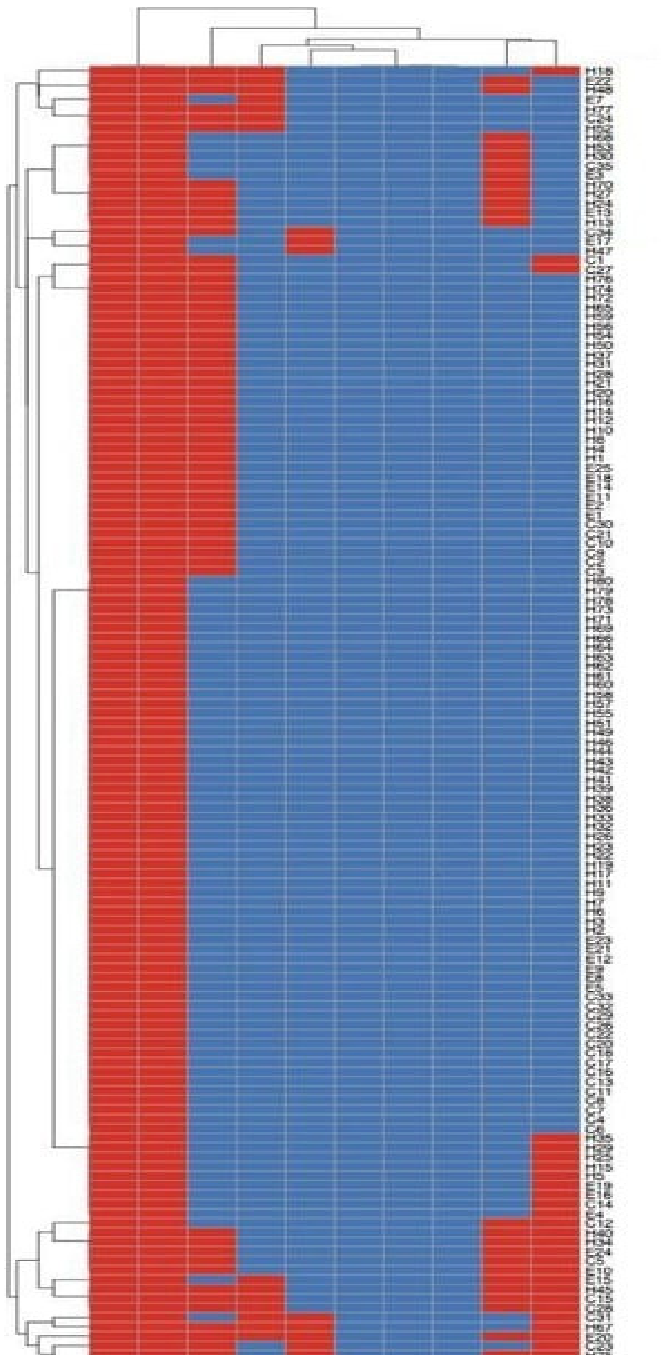

Figure S3: Heat map showing the occurrence of virulence genes in the studied diarrheagenic *E. coli* isolates. In the heat map, red and blue colors refer to the presence and absence of a particular virulence gene, respectively. The dendogram represents the hierarchical clustering of the isolates and the virulence genes. The code numbers on the right of the heat map refer to the isolate numbers from human (H), cow (C) and equine (E) sources.

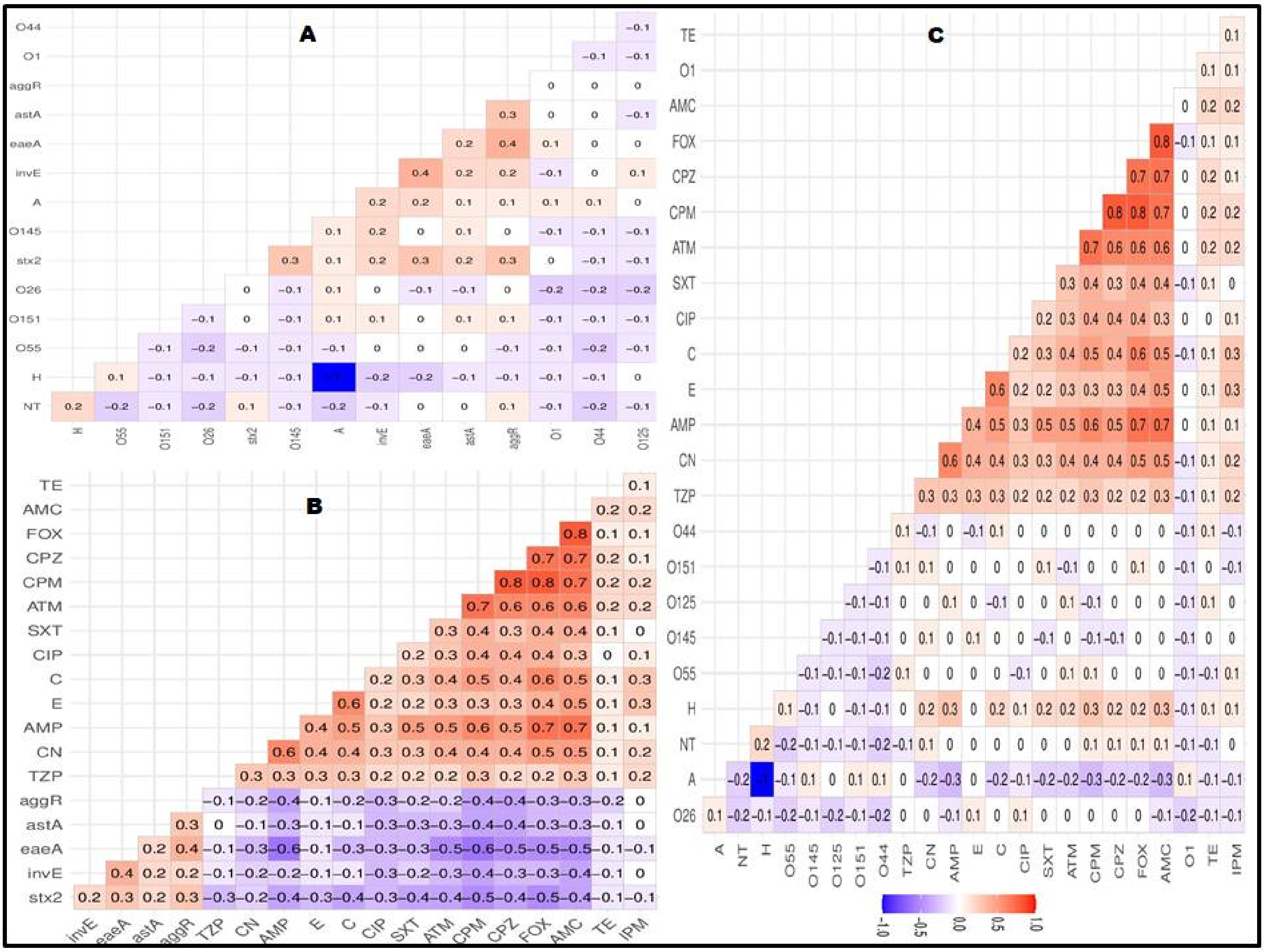

Figure S4. Correlation coefficient values (R) between serotypes and virulence genes (A), antimicrobial resistance and virulence genes (B) and serotypes and antimicrobial resistance (C) of diarrheagenic *E. coli*. Genes encoding for adhesion (*fim*H and *kps*M), enterotoxin (*ast*A), intimin (*eae*A), Vero toxins (*vtx*), Shiga toxins (*stx*), hemolysin (*hly*), outer membrane protein (*omp*) and transcriptional activators for aggregative adherence fimbriae (*aggR*) and invasion (*invE*) genes. H; human, A; animal, AMP: ampicillin, AMC: amoxicillin/clavulanic acid, ATM: aztreonam, FOX: cefoxitin, CPZ: cefoperazone, CPM: cefepime, TZP: piperacillin/tazobactam, IPM: imipenem, C: chloramphenicol, E: erythromycin, CN: gentamycin, CIP: ciprofloxacin, SXT: sulfamethoxazole/trimethoprim, TE: tetracycline.
